# Research Design Processes in Serious Games for Adolescent Mental Health: Systematic Review

**DOI:** 10.2196/77173

**Published:** 2026-04-10

**Authors:** S T Rooij, de, D A Kuipers, J T Prins, J P E N Pierie

**Affiliations:** 1 Lectorate of Design Driven Innovation, School of Design, NHL Stenden University of Applied Sciences, Rengerslaan 8-10, Leeuwarden, 8917 DD, The Netherlands, 31 088 991 7000; 2 Wenckebach Institute, University Medical Center Groningen, Groningen, The Netherlands; 3 Faculty of Medical Sciences, University Medical Center Groningen, Groningen, The Netherlands

**Keywords:** serious games, serious gaming, adolescent mental health, learning transfer, boundary crossing, model of reality, design rationale, game-based learning, health care innovation

## Abstract

**Background:**

Serious games are increasingly recognized as effective tools in adolescent mental health interventions, providing engaging platforms for emotional regulation, skill development, and behavioral change. However, the ways in which core theoretical concepts such as transfer, boundary crossing, and models of reality are incorporated into serious game designs are not consistently described in the literature. Clarifying how these concepts are addressed is important for understanding how game-based learning may connect to real-world health care practice.

**Objective:**

This systematic review aims to examine how serious games for adolescent health care are designed to support learning and facilitate outcomes. Specifically, it examines how the design incorporates constructs of transfer, boundary crossing, and models of reality, and how these elements are represented across published studies.

**Methods:**

We conducted a systematic search across 5 databases (PubMed, Scopus, ERIC, PsycINFO, and EMBASE) covering publications up to 2023. Studies were included if they involved serious games targeting adolescents with behavioral or developmental health concerns. Titles and abstracts were screened independently by 2 reviewers, with disagreements resolved by a third party. A qualitative analytical framework was applied to identify elements of design, with a particular focus on transfer, boundary crossing, and models of reality.

**Results:**

Thirty-three studies met the inclusion criteria. Figural transfer was identified in 24 studies, while literal transfer was identified in 10 studies. Among boundary-crossing mechanisms, reflection occurred most frequently (22 studies), whereas transformation was observed in 3 studies. Causal and procedural models of reality were most commonly identified as primary model types, whereas relational and structural models were more often reported as secondary. Explicit design rationales were infrequently reported across studies.

**Conclusions:**

This review demonstrates that serious games for adolescent mental health most frequently emphasize reflective and representational forms of learning. Across the reviewed studies, theoretical constructs related to transfer, boundary crossing, and models of reality were often implicitly embedded rather than explicitly articulated. The proposed analytical framework offers a structured approach for analyzing these design characteristics and may support designers, researchers, and health care professionals in more explicitly aligning design choices with intended learning mechanisms and real-world applications.

## Introduction

### Context and Rationale

Playing video games is an integral part of adolescent culture, serving not only as entertainment but also as a platform for skill development and identity exploration [1]. For adolescents with behavioral or developmental needs, gaming provides structured environments to practice competencies that support personal and social growth. Therapeutic games can enhance emotional regulation, problem-solving, and executive function, making them valuable for interventions addressing these challenges [2]. Health care professionals increasingly explore how these skills can be applied in therapeutic contexts, particularly through digital interventions that expand access to psychological support [3]. However, further research is needed to understand how in-game learning effectively transfers to real-world applications and how game design supports this process.

A growing body of research examines the role of serious games in mental health interventions. Systematic reviews and meta-analyses have shown their effectiveness in addressing anxiety [4], depression [5,6], emotional regulation [7], and broader psychological well-being [8,9]. Serious games targeting adolescents show particular promise for fostering engagement and skill development among populations who may resist traditional forms of therapy [10]. Yet, existing reviews primarily evaluate outcomes rather than examining how their design rationale enables learning and behavioral change. Recent work has begun to highlight the need for design-focused evaluation frameworks in serious gaming interventions [11], including the integration of psychological, motivational, and contextual design principles [12]. Understanding these mechanisms is critical for developing scalable, evidence-based interventions that integrate meaningfully into health care practice.

### Health Care and Serious Gaming

Adolescents with behavioral or developmental challenges often engage deeply with video games, offering both opportunities for therapeutic skill development and challenges for implementation in health care contexts [13]. Many abilities cultivated through gameplay, such as executive functioning, emotional regulation, and social communication, align closely with health care objectives [14]. Serious games, distinct from entertainment games, are intentionally designed with educational or therapeutic intent to foster learning and behavioral change through structured feedback and interaction [15].

For adolescents experiencing anxiety, social stress, or executive dysfunction, game environments provide opportunities for cognitive rehearsal and emotional self-regulation [16]. The structured and controllable nature of gameplay can reduce stress and facilitate gradual exposure, particularly among individuals with autism spectrum disorder [17]. Integrating biofeedback features, such as heart rate monitoring, further enhances awareness of physiological states and supports self-management [3]. Multiplayer and cooperative formats extend the therapeutic potential by promoting communication and collaboration, aligning with broader educational and clinical goals [18].

From a motivational perspective, serious gaming, or the process of applying game-based interaction in structured interventions, benefits from self-determination theory, emphasizing autonomy, competence, and relatedness [19]. Well-designed games can support all 3 dimensions, fostering intrinsic motivation and engagement. Because players differ in what motivates them, adaptable and inclusive designs are essential [20]. Despite growing evidence for their value, the integration of serious games into health care remains limited. Participatory design, involving adolescents, caregivers, and clinicians, can enhance contextual relevance and therapeutic alignment [21]. In this sense, serious games can function as mediating tools that connect education, therapy, and everyday life, establishing conditions for what boundary-crossing theory [22] describes as transitions between distinct domains of practice.

### Boundary Crossing and Learning Mechanisms

Serious games designed for therapeutic or educational purposes often simulate or abstract real-world challenges to foster learning and behavioral change [23]. Understanding how these games enable the application of skills beyond gameplay remains complex [24,25]. Drawing on model-based reasoning, effective learning occurs when interactive representations balance abstraction with real-world fidelity, enabling players to engage in both conceptual exploration and situated practice [26,27]. Within this context, serious games can act as boundary objects or artifacts that mediate between distinct domains such as health care, education, and daily life [28].

Boundary crossing theory helps explain how serious games facilitate transitions between these domains. Akkerman and Bakker [22] identify 4 mechanisms of boundary crossing: identification, coordination, reflection, and transformation, which enable knowledge movement across contexts. In serious games, these mechanisms appear when players assume new roles, collaborate across disciplines, or reflect on in-game experiences to link them with real-world contexts [29]. Through these processes, players act as boundary crossers, integrating insights, perspectives, and strategies from simulated environments into their personal or professional realities.

Designing for boundary crossing requires balancing structure and flexibility. Overly standardized mechanics can constrain learning, while excessive abstraction risks disconnecting gameplay from therapeutic goals. Scaffolding methods, such as reflective dialogue, adaptive feedback, and structured debriefing, can strengthen the link between play and practice [30,31]. When aligned with health care objectives, serious games can thus serve as both learning tools and vehicles for systemic integration [32].

### Transfer and Model of Reality

The ability to apply knowledge and skills from one context to another is central to the educational and therapeutic values of serious games [33,34]. In health care, successful transfer determines whether insights gained during gameplay lead to meaningful behavioral or cognitive changes in real-world settings [35,36]. Two complementary types of transfer are often distinguished: literal transfer, which involves the direct application of learned skills in similar contexts, and figural transfer, which requires abstraction and adaptation to new or unfamiliar situations [33]. While literal transfer supports practice through realism, figural transfer fosters generalizable learning and adaptability [34].

Boundary crossing and transfer are interdependent. Mechanisms such as reflection and coordination provide the cognitive scaffolding that allows experiences within a game to extend beyond its boundaries. Thus, serious games do not merely teach within a virtual system but prepare players to act beyond it.

The model of reality represented in a game further defines how transfer occurs. Each serious game embodies a representational model, causal, relational, procedural, or structural, that shapes how aspects of the real world are simplified or transformed during play. As research by Wenzler [37] notes, these models mediate the relationship between simulated and actual experiences, influencing both fidelity and interpretive depth. Complementing this, the Bogost [38] concept of procedural rhetoric highlights how the logic of rules and mechanics communicates meaning, shaping how players understand complex systems. Together, these frameworks explain how serious games model reality in ways that make transfer possible and meaningful.

For adolescents, such design choices are crucial. Games provide safe environments for exploring identity, practicing regulation, and testing social behaviors [39,40]. By combining representational fidelity with reflective abstraction, serious games can translate in-game learning into durable real-world competencies [41].

### Aim and Research Questions

This systematic review examines how serious games in health care incorporate the constructs of transfer, boundary crossing, and models of reality within their design rationale and implementation. Unlike prior reviews that focus on outcomes, this study analyzes the conceptual coherence of design approaches, identifying how theoretical frameworks inform game development and affect the connection between simulated and real-world practice.

The study aims to clarify how design choices facilitate learning transfer, enable boundary-crossing conditions across educational and clinical domains, and represent reality through varying degrees of abstraction and fidelity.

We proposed the following research questions for this study:

- RQ1: To what extent do publications on serious games in health care incorporate design elements related to learning transfer, boundary crossing, and models of reality?
- RQ2: How are these 3 constructs conceptualized and operationalized within the design and development of serious games for health care?
- RQ3: What conceptual or methodological gaps remain in current design rationales, and how might addressing them strengthen the translational potential of serious games in health care?

## Methods

This systematic review followed the PRISMA (Preferred Reporting Items for Systematic Reviews and Meta-Analyses) 2020 [42] guidelines to ensure transparency and reproducibility (Checklist 1). The review was not registered beforehand. The study focused on identifying serious game artifacts explicitly used in adolescent health care relationships. These artifacts were defined as intentionally designed games aimed at supporting learning or behavioral change rather than entertainment or measurement tools.

Five databases (PubMed, Scopus, ERIC, PsycINFO, and EMBASE) were searched for potentially relevant studies. The databases were selected for their combined coverage of health, psychology, and social sciences. Search strategies were developed in consultation with an information specialist and refined through several trial searches to improve precision and comprehensiveness (see Multimedia Appendix 1 for search keys). Searches included papers published from database inception until October 2023 and were conducted between April 3 and May 21, 2023.

The search strategy was built around 4 intersecting conceptual domains to ensure relevant publications: (1) the health care domain, including games addressing therapeutic, preventive, or educational objectives; (2) the serious game domain, which included artifacts designed with explicit learning intent; (3) the problem domain, referring to behavioral, emotional, or developmental challenges; and (4) the target audience domain, referring to adolescents in health-related contexts. Only studies located within the overlap of all 4 domains were included. This approach yielded 3997 records, which were reduced to 2296 after duplicate removal. The broader search and the choice in strategy to try to find these artifacts were intentional. As a framework for evaluating the design rationale of serious games does not yet exist, we expected any data to be implicit.

To systematically evaluate design rationale and learning mechanisms, a structured framework was applied as follows. Artifact identification, which identifies the serious game, its name, and targeted skills or competencies [43,44]; Objective, which identifies the intent of the artifact by describing the desired learning process; Boundary crossing, which assesses how the game functions as a boundary object, supporting learning mechanisms’ identification, coordination, reflection, or transformation [29]; Transfer, which evaluates how learning extends from game to reality, distinguishing between literal and figural transfer [33]; and Model of reality, which categorizes how the game represents real-world systems through 4 types: causal, relational, procedural, and structural models.

To ensure consistency and reliability, the framework was pilot-tested on a subset of 5 randomly selected studies before full application. Both reviewers applied the criteria independently, after which definitions were refined through consensus.

For coding consistency, categories were defined as follows. Literal transfer refers to games that replicate real-world tasks or environments, enabling direct practice of target behaviors. Figural transfer refers to games using symbolic or metaphorical representations that require players to generalize learning beyond the game. Boundary-crossing mechanisms were coded according to Akkerman and Bakker’s [22] framework: identification (recognizing new roles or perspectives), coordination (managing interactions across tasks or roles), reflection (linking in-game experiences to real-world contexts), and transformation (applying learning across contexts in ways that alter practice). Models of reality were categorized as causal (cause-effect logic), procedural (stepwise processes or rules), relational (social or interpersonal dynamics), or structural (conceptual or systemic relationships).

## Results

### Screening Process

Two reviewers independently screened the 2296 titles and abstracts using Rayyan (Qatar Computing Research Institute). Studies were excluded if they focused on simulation for data modeling, used games only as measurement tools (eg, eye-tracking or motor-tracking), and lacked peer review or methodological detail. Studies were included when they addressed a health care–related challenge affecting adolescents’ social, emotional, cognitive, or developmental functioning and used a serious game as an active learning or therapeutic tool. Because many included populations do not share a single diagnostic label, both health care and the target audience were interpreted more broadly. Disagreements were resolved through a structured consensus process and by including a third reviewer.

**Figure 1.**
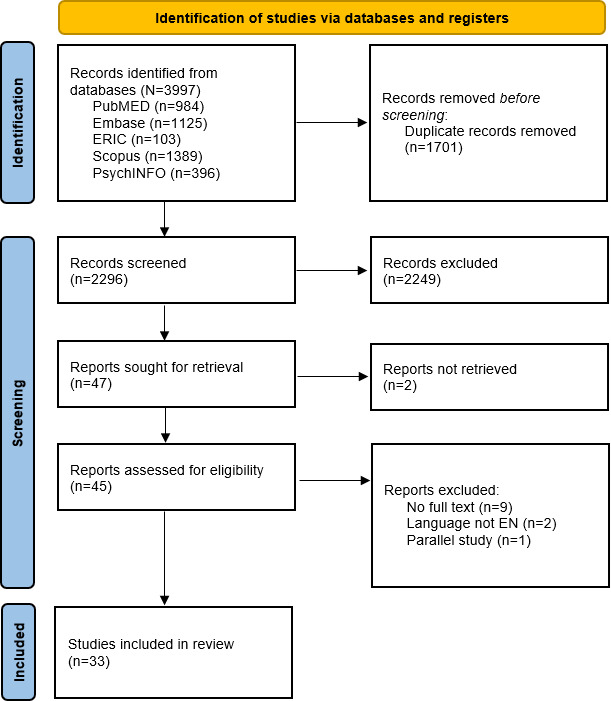
Selection process for review: PRISMA (Preferred Reporting Items for Systematic Reviews and Meta-Analyses) 2020 flow diagram.

Following this stage, 47 papers were reviewed in full, resulting in 33 included studies. An overview of the key characteristics of the 33 included studies is provided in Table 1 (see Table 2 for the coding table). The excluded papers comprised 9 conference abstracts without accompanying full papers, 2 unavailable manuscripts, 2 available only in French, and 2 publications about the same artifact and context. A PRISMA 2020 flow diagram (Figure 1) summarizes the study selection process from 3997 initial records to 33 included studies.

**Table 1.** Characteristics of the included studies (N=33)*.*

Author and year	Title	Population (n; age range, y)	Study design	Health domain	Context
David et al (2022) [18]	Do improvements in therapeutic game-based skills transfer to real-life improvements in children’s emotion-regulation abilities and mental health?	Children and adolescents (48; 10‐16 y)	Feasibility/pilot study (secondary analysis of RCT^a^ data)	Emotion regulation, resilience, and mental health	Digital game intervention in clinical/therapeutic context
Zhang et al (2018) [45]	Understanding performance and verbal-communication of children with ASD in a collaborative virtual environment	Children with ASD^b^ and typically developing peers (28; NR^c^)	Feasibility/pilot study	Social communication in ASD	Collaborative virtual environment/lab
Bossavit and Parsons (2018) [46]	Outcomes for design and learning when teenagers with autism codesign a serious game: a pilot study	Teenagers with ASD (6; 11‐15 y)	Feasibility/pilot study (participatory co-design)	Teamwork, social interaction, and learning design	School/co-design setting
Beach and Wendt (2015) [47]	Social interaction development through immersive virtual environments	Adolescents with high-functioning ASD (2; 15‐18 y)	Qualitative pilot case study	Social interaction development	Immersive VR^d^/educational context
Wang and Xing (2022) [48]	Supporting youth with autism learning social competence: a comparison of game- and nongame-based activities in 3D virtual world	Adolescents with ASD (11; NR)	Comparative study	Social competence	3D virtual world/educational setting
Ke and Moon (2018) [49]	Virtual collaborative gaming as social skills training for high-functioning autistic children	Children with high-functioning ASD (8; 10‐14 y)	Mixed method multiple case study	Social skills training	Virtual playground/online environment
Kee et al (2022) [50]	Virtual reality-based social skills training for children with autism spectrum disorder	Children with ASD (7; 10‐14 y)	Single-case intervention study	Social communication skills	Desktop VR/clinical or educational context
Johnson et al (2022) [51]	Charisma™ virtual social training: a digital health platform and protocol	Children and adolescents with social difficulties (67; 9‐17 y)	Feasibility/pilot intervention study	Social coaching and social cognition	Remote/hybrid digital health settings
Atherton and Cross (2021) [52]	The use of analog and digital games for autism interventions	Autistic individuals across reviewed studies (NR; NR)	Scoping review	Cognitive and social skills in ASD	Mixed analog/digital intervention contexts
Thomsen and Adjorlu (2021) [53]	A collaborative virtual reality supermarket training application to teach shopping skills to young individuals with autism spectrum disorder	Adolescents with ASD (8; NR)	Feasibility/pilot study	Daily living skills (shopping and money management)	Collaborative VR supermarket environment
Vallefuoco et al (2022) [54]	Design of a serious game for enhancing money use in teens with autism spectrum disorder	Target group: adolescents with ASD (NR; 13‐19 y)	Design and development study	Money use and financial literacy	3D digital game, likely school/therapy linked
Caria et al (2018) [55]	The design of web games for helping young high-functioning autistics in learning how to manage money	Adolescents and young adults with high-functioning ASD (6; NR)	Design and usability study	Practical money management	Web-based games in educational context
Ringland et al (2017) [56]	Making in Minecraft: a means of self-expression for youth with autism	Autistic children and youth in an online community (NR; NR)	Qualitative ethnographic study	Self-expression, creativity, and participation	Minecraft-based online/club settings
Kandalaft et al (2013) [57]	Virtual reality social cognition training for young adults with high-functioning autism	Young adults with high-functioning ASD (8; 18‐26 y)	Intervention study (pre–post with follow-up)	Social cognition	VR lab/clinical research settings
Stichter et al (2014) [58]	iSocial: delivering the social competence intervention for adolescents (SCI-A) in a 3D virtual learning environment for youth with high functioning autism	Adolescents with high-functioning ASD (NR; 12‐17 y)	Intervention study (pre–post)	Social competence	Virtual learning environment
Lahiri et al (2011) [59]	Design of a virtual reality based adaptive response technology for children with autism spectrum disorder	Adolescents with ASD (6; NR)	Design and usability study	Social communication/response to social cues	VR-based system in lab/clinical settings
Grynszpan et al (2007) [60]	Exploring the influence of task assignment and output modalities on computerized training for autism	Children with ASD and typically developing peers (NR; NR)	Experimental training study	Executive functions and communication	Computer-based training environment
García-Redondo et al (2019) [61]	Serious games and their effect improving attention in students with learning disabilities	Students with learning disabilities (NR; primary school age)	Intervention study	Attention and executive functioning	School-based game use
Kerns et al (2017) [62]	Attention and working memory training: a feasibility study in children with neurodevelopmental disorders	Children with neurodevelopmental disorders (NR; 6‐13 y)	Feasibility/pilot study	Attention and working memory	Educational/clinical training context
Ghanouni et al (2020) [63]	Design elements during development of videogame programs for children with autism spectrum disorder: Stakeholders’ viewpoints	Stakeholders incl. youth with ASD (26; youth 13‐17 y)	Qualitative study	Design elements for ASD game interventions	Stakeholder workshops/design sessions
Dovis et al (2015) [64]	Improving executive functioning in children with ADHD: training multiple executive functions within the context of a computer game. a randomized double-blind placebo controlled trial	Children with ADHD^e^ (89; 8‐12 y)	RCT	Executive functioning	Home/clinical digital training
Lahiri et al (2011) [65]	Design of a gaze-sensitive virtual social interactive system for children with autism.	Adolescents with ASD (6; NR)	Design and usability study	Social gaze and social communication	VR system in lab/clinical context
MacCormack and Freeman (2019) [66]	Part 2: the virtual environment social program for youths with autism spectrum disorder	Children with ASD (4; 11‐13 y)	Feasibility/pilot intervention study	Social competence and play therapy	Clinic/community-based virtual program
Wang et al (2017) [67]	Fostering verbal and non-verbal social interactions in a 3D collaborative virtual learning environment: a case study of youth with autism spectrum disorders learning social competence in iSocial	Youth with ASD (11; NR)	Exploratory case study	Verbal and nonverbal social interaction	3D collaborative virtual learning environment
Lerman et al (2017) [68]	A clinic-based assessment for evaluating job-related social skills in adolescents and adults with autism	Adolescents and adults with ASD (8; 16‐32 y)	Assessment study	Vocational social skills	Clinic-based simulated workplace
Elhaddadi et al (2021) [69]	Serious games to teach emotion recognition to children with autism spectrum disorders (ASD)	Children and adolescents with ASD (32; NR)	Intervention study (pre–post)	Emotion recognition	Computer-based/school or lab context
Amat et al (2021) [70]	Design of an interactive virtual reality system, InViRS, for joint attention practice in autistic children	Children with ASD and typically developing peers (18; NR)	Design and feasibility study	Joint attention practice	VR-based interactive system
Bossenbroek et al (2020) [71]	Efficacy of a virtual reality biofeedback game (DEEP) to reduce anxiety and disruptive classroom behavior: single-case study	Adolescents in special secondary education (8; mean≈15, SD 1.83 y)	Single-case experimental design	Anxiety reduction and emotion regulation	VR biofeedback game in school/clinical context
Kahlon et al (2019) [72]	Virtual reality exposure therapy for adolescents with fear of public speaking: a non-randomized feasibility and pilot study	Adolescents with public speaking anxiety (27; 13‐16 y)	Feasibility/pilot study	Social anxiety/fear of public speaking	VR exposure in clinical settings
Lahiri et al (2015) [73]	A physiologically informed virtual reality based social communication system for individuals with autism	Adolescents with ASD and typically developing peers (12; mean≈15.9, SD 2.15 y)	Usability and proof-of-concept study	Social communication and engagement	VR-based social communication system
Fornasari et al (2013) [74]	Navigation and exploration of an urban virtual environment by children with autism spectrum disorder compared to children with typical development	Children with ASD and typically developing peers (32; 7‐14 y)	Comparative experimental study	Urban navigation and spatial behavior	Virtual urban environment/lab
Ringland (2019) [75]	“Autsome”: fostering an autistic identity in an online Minecraft community for youth with autism	Autistic children and youth in online community (NR; NR)	Qualitative ethnographic study	Autistic identity and social belonging	Online Minecraft community
Finke et al (2018) [16]	“To be quite honest, if it wasn’t for videogames I wouldn’t have a social life at all”: motivations of young adults with autism spectrum disorder for playing videogames as leisure	Young adults with ASD (10; 18‐24 y)	Qualitative interview study	Leisure gaming, social life, and identity	Home/leisure gaming contexts

a RCT: randomized controlled trial.

b ASD: autism spectrum disorder.

c NR: not reported.

d VR: virtual reality.

e ADHD: attention-deficit/hyperactivity disorder.

**Table 2.** Overview of design characteristics and analytical coding of included studies*.*

Author and year	Artifact	Objective	Learning mechanism	Transfer type	Model type
David et al (2022) [18]	REThink: emotional regulation and resilience for adolescents	Based on REBT^a^; structured levels for emotion and cognition	Identification and reflection emphasized	Figural transfer. psychological fidelity	Relational, causal: REBT framework modeling emotional-cognitive feedback loops
Zhang et al (2018) [45]	CVE^b^: communication and collaboration skills for ASD^c^ children	FSM^d^-based; real-time feedback for dynamic collaboration	Identification and coordination mechanisms present	Figural transfer. psychological fidelity via metaphorical social interaction scenarios	Causal, relational: FSM-based system structuring collaboration through cause–effect interactions
Bossavit and Parsons (2018) [46]	Geography-themed game: teamwork and geography knowledge for ASD adolescents	3T sandwich model; participatory design with tailored interaction modes	Identification and coordination in team-based play	Figural transfer: psychological fidelity with functional teamwork mechanics	Relational, procedural: team-based collaboration modeled through iterative design-feedback cycles
Beach and Wendt (2015) [47]	Immersive virtual environment: social interaction training for ASD students	Customized VR^e^ scenarios based on social challenges	Identification and reflection mechanisms utilized	Figural transfer: psychological fidelity supporting skills transfer	Procedural, relational: guided VR social sequences replicating real-world interaction flows
Wang and Xing, (2022) [48]	3D virtual world game: collaborative social skill training for ASD youth	Scaffolded 3D gameplay; adaptive learning mechanics	Coordination through collaborative gameplay; reflection on interaction patterns	Figural transfer: fidelity via adaptive gameplay scenarios	Causal, relational: behavioral frameworks linking feedback to adaptive social competence
Ke and Moon, (2018) [49]	3D virtual playground: competition, role-play, and design for social interaction	Constructed in OpenSimulator; learner-adaptive	Identification via role-play and task adaptation	Figural transfer: physical fidelity to real-world context	Causal, procedural: role-play tasks reinforcing behavior through structured adaptation loops
Ke et al (2022) [50]	Desktop VR social skills program: social communication for ASD children	Scenario-based role-play; multimodal feedback	Coordination and reflection through tasks	Figural transfer from simulated to real	Procedural, relational: scenario-based communication tasks following stepwise learning processes
Johnson et al (2022) [51]	CHARISMA-VST^f^: low-immersion VR for pediatric social skill training	Strength-based coaching; remote-friendly	Reflection through peer role-play interactions	Figural transfer: psychological fidelity	Relational, causal: peer role-play and coaching systems modeling social cognition and feedback
Atherton and Cross (2021) [52]	Gamified interventions: digital and analog games for social and cognitive skills	Behavioral reinforcement; adaptive narratives	Reflection via joint engagement tasks	Figural transfer: blended fidelity with narrative integration	Causal, structural: cognitive-behavioral feedback integrated into narrative skill frameworks
Thomsen and Adjorlu (2021) [53]	VR supermarket training: shopping and money management for ASD adolescents	Co-designed with teachers; task-based learning	Coordination and transformation via tasks	Literal transfer: physical and psychological fidelity	Procedural, causal: shopping and money management simulated through task-based learning loops
Vallefuoco et al (2021) [54]	€UReka: 3D game for recognizing and handling money	Participatory design with multidisciplinary input; tailored challenges	Identification via realistic tasks; reflection through interactive learning	Literal transfer: psychological fidelity	Procedural, structural: iterative money-handling training structured within financial skill frameworks
Caria et al (2018) [55]	Web-based games for practical money management	Iterative co-design process; accessibility focus	Coordination via financial decision-making tasks	Literal transfer: psychological fidelity	Procedural, structural: decision-making tasks organized through educational usability structures
Ringland et al (2017) [56]	Minecraft-based activities for creativity and self-expression	Creative focus; adaptive gameplay for needs	Reflection through collaboration and shared space	Figural transfer: psychological fidelity via metaphorical engagement	Relational, structural: maker culture modeling social creativity within conceptual frameworks
Kandalaft et al (2013) [57]	VR-SCT^g^: virtual reality-based training for social cognition	Immersive VR; real-life scenario simulation	Reflection on cognition; transformation via practice	Figural transfer: psychological fidelity aligning with real skills	Relational, procedural: VR social cognition training reflecting interpersonal and sequential skill use
Stichter et al (2014) [58]	iSocial 3D VLE^h^: social competence training for adolescents	ABA^i^ principles in collaborative virtual environments	Coordination and reflection via guided interaction	Figural transfer: psychological and physical fidelity for social skills	Causal, relational: ABA principles linking stimuli and responses within collaborative learning
Lahiri et al (2011) [59]	VR system for social communication tasks for ASD children	Real-time eye-gaze monitoring with adaptive tasks	Identification and reflection with social avatars	Figural transfer: psychological fidelity	Causal, procedural: adaptive gaze monitoring creating real-time feedback-based learning sequences
Grynszpan et al (2007) [60]	Multimodal games to enhance executive functions in ASD	Multimodal testing; task differentiation by domain	Coordination of spatial and pragmatic skills	Literal transfer: psychological fidelity tailored to user training	Structural, procedural: executive functions structured conceptually and trained through procedural tasks
García-Redondo et al (2019) [61]	Games based on the Gardner multiple intelligences for attention training	Narrative and cognitive reinforcement strategies	Reflection on cognitive strengths and interactive tasks	Figural transfer: psychological fidelity through narratives	Structural, causal: multiple intelligences mapped to causal reinforcement mechanisms for attention
Kerns et al (2017) [62]	Caribbean Quest: game-based attention and memory training	Metacognitive strategies integrated with gameplay	Reflection and transformation of cognitive skills	Figural and literal transfer via adaptive training: psychological fidelity	Structural, procedural: cognitive remediation organizing mental functions into adaptive training sequences
Ghanouni et al (2020) [63]	Stakeholder-informed videogame programs for ASD	Participatory design with stakeholder feedback	Reflection via user-centered design processes	Figural transfer for adaptable social skills: psychological fidelity	Relational, structural: stakeholder-based co-design modeling social systems within structured frameworks
Dovis et al (2015) [64]	Braingame Brian: game targeting multiple executive functions in ADHD^j^	Gamified EF^k^ training integrating WM^l^, inhibition, and flexibility	Coordination through tasks; reflection on performance	Figural transfer: psychological fidelity	Structural, causal: executive functions integrated into structured cognitive frameworks with feedback
Lahiri et al (2011) [65]	VIGART^m^: VR-based system for real-time gaze interaction	Dynamic gaze-based feedback for social communication	Reflection and identification with gaze data	Literal transfer: physical fidelity	Causal, procedural: real-time gaze-based feedback linking attention to behavioral reinforcement
MacCormack and Freeman (2019) [66]	Minecraft-based structured/free play for social competence in ASD	Peer mediation, video modeling, and structured play methods	Coordination through mediated interactions	Figural transfer through gameplay scenarios	Relational, procedural: peer mediation and structured play modeling social competence through sequences
Wang et al (2017) [67]	iSocial: 3D CVLE^n^ for ASD social competence	Narrative-embedded, goal-oriented, peer-supported tasks	Reflection in collaborative 3D environments	Figural transfer for social behaviors: psychological fidelity	Causal, relational. behavioral frameworks reinforcing social interaction in 3D virtual collaboration
Lerman et al (2017) [68]	Protocol for job-related social skill assessment in ASD	Simulated workplace tasks for skill assessment	Identification in work-simulated environments	Literal transfer of job-related skills: physical and psychological fidelity	Procedural, causal: simulated workplace tasks reflecting vocational training processes
Elhaddadi et al (2021) [69]	Multisensory game for teaching emotion recognition in ASD	Adaptation to cultural context and emotion-based tasks	Reflection through emotion recognition tasks	Literal transfer of emotion skills: psychological fidelity	Causal, structural: emotion recognition modeled through behavioral and cultural task frameworks
Amat et al (2021) [70]	VR-based game for gaze sharing and following in ASD	Real-time gaze tracking with adaptive feedback	Coordination through gaze-based interaction	Literal transfer via gaze coordination: physical fidelity	Causal, procedural: gaze-tracking feedback driving joint attention in sequential learning loops
Bossenbroek et al (2020) [71]	VR biofeedback game for anxiety and disruptive behavior reduction	Diaphragmatic breathing integrated with biofeedback	Reflection through self-awareness in VR	Figural transfer: psychological fidelity for emotion regulation	Causal, procedural: biofeedback linking physiological regulation to emotional response mechanisms
Kahlon et al (2019) [72]	VR exposure therapy simulating public speaking scenarios	Age-appropriate scenarios for exposure therapy	Reflection via structured exposure tasks	Figural transfer in social performance: psychological fidelity	Procedural, causal: exposure therapy modeled through sequential desensitization and behavioral outcomes
Lahiri et al (2015) [73]	VR-based system using physiological engagement for social communication	Adaptive response systems using engagement metrics	Reflection and coordination with biofeedback	Figural transfer tied to engagement cues: psychological fidelity	Causal, procedural: physiological feedback loops structuring adaptive engagement tasks
Fornasari et al (2013) [74]	Virtual environment simulation for urban navigation	Simplified navigation tasks for ASD; comparative evaluation	Reflection through exploration tasks in VE	Literal transfer of urban navigation skills: physical fidelity	Structural, procedural: simplified urban environments representing spatial systems for navigation practice
Ringland (2019) [75]	Minecraft-based online community for fostering autistic identity	Inclusive community promoting identity and safety	Coordination via shared gaming and identity	Figural transfer, psychological fidelity in building resilience	Relational, structural: online community fostering identity within structured digital interaction systems
Finke et al (2018) [16]	Videogaming as leisure activity for social and identity development	Leisure gaming to enhance social and communicative skills	Reflection on social experiences in gaming	Figural transfer: psychological fidelity emphasizing engagement	Relational, structural: gaming as social-identity context framed by interactional structures

a REBT: rational emotive behavior therapy.

b CVE: collaborative virtual environment.

c ASD: autism spectrum disorder.

d FSM: finite state machine.

e VR: virtual reality.

f CHARISMA-VST: charisma virtual social training.

g VR-SCT: virtual reality social cognition training.

h 3D-VLE: 3D virtual learning environment.

i ABA: applied behavior analysis.

j ADHD: attention-deficit/hyperactivity disorder.

k EF: executive functions.

l WM: working memory.

m VIGART: virtual interactive gaze-based adaptive response technology.

n CVLE: collaborative virtual learning environment.

Inter-rater reliability for the initial round of coding was κ=0.55, which reflects moderate agreement (κ=0.41‐0.60) [76,77]. Moderate agreement is acceptable in qualitative content analyses that require inferential judgment, particularly when coding implicit or theory-driven categories, such as transfer types, boundary-crossing mechanisms, and model typologies. These constructs were often not explicitly described, making some variability in coder interpretation expected.

### Transfer

Many games prioritized figural transfer (n=24), using metaphorical or symbolic representations to promote generalizable cognitive and emotional learning. Only 10 studies utilized literal transfer, replicating real-life tasks or environments. Examples include virtual reality supermarket training [53], which simulates grocery shopping to teach money management skills; Braingame Brian [64], which trains executive functions through structured gameplay; figural approaches, such as DEEP [71], which teaches emotional regulation through metaphorical underwater immersion; and iSocial [58], which develops social competence using virtual role-play, illustrating a focus on reflective learning rather than direct behavioral replication.

Despite these efforts, few studies incorporated explicit scaffolding or debriefing mechanisms to reinforce transfer, and longitudinal evaluations were rare. While short-term improvements in executive function and social skills were frequently measured, few studies investigated whether these abilities persisted or generalized beyond the intervention [72]. The overall dominance of figural transfer suggests that most games aim to develop reflective and conceptual understanding rather than direct behavioral transformation.

### Boundary Crossing

The distribution of learning mechanisms was uneven: reflection appeared in 22 studies, coordination in 13, identification in 9, and transformation in 3. This distribution shows that most games promote self-awareness and perspective taking but rarely enable systemic changes or real-world applications. For instance, Minecraft-based communities [78] provided safe spaces for adolescents to explore identity and social belonging, while DEEP [71] encouraged users to monitor and regulate emotions. In contrast, CHARISMA-virtual social training [51] exemplified stronger coordination and transformation, linking coaching-based gameplay with clinical follow-up and remote engagement. While participatory design processes frequently involved caregivers, therapists, or educators, few studies explicitly articulated a boundary-crossing framework. Consequently, most serious games functioned as standalone interventions, rather than integrated components within health care systems.

### Models of Reality

The conceptualization of models of reality varied significantly across the reviewed studies. Based on an analytical typology developed for this review, 4 types were identified: causal (n=12), procedural (n=7), relational (n=9), and structural (n=5). Causal and procedural models were most frequent overall (each appearing 19 times across primary and secondary codings), reflecting a focus on feedback-driven learning and stepwise behavioral training. Relational and structural models typically appeared as secondary logics, emphasizing social interaction and conceptual scaffolding. Common examples include virtual reality exposure therapy [72], which applied a causal–procedural model to replicate public speaking scenarios, and iSocial [58], which integrated relational and structural logics to foster social competence through collaborative virtual environments. In contrast, some games oversimplified real-world complexity, such as urban navigation simulators [74], which lacked the unpredictability representative of real-life settings.

### Alignment Between Model Type and Transfer

A cross-analysis of model types and transfer forms revealed strong internal alignment between theoretical constructs and design intent. Causal–figural (n=9) and relational–figural (n=9) pairings dominated, representing enabling configurations that support reflective and metaphorical learning. Conversely, procedural–literal (n=4) and structural–literal (n=2) combinations were less common but corresponded to direct skill training and conceptual knowledge transfer.

These results indicate that most games intentionally (or implicitly) align their learning mechanisms with their representational logic: figural models enable reflective and identity-building experiences, while literal designs facilitate direct behavioral rehearsal. The absence of relational–literal pairings further underscores that social learning is almost exclusively represented symbolically in adolescent mental health games.

### Stakeholder Engagement

Stakeholder engagement was a consistent strength across many studies, ensuring relevance and usability through participatory design. For example, €UReka [54], a 3D game for financial literacy, involved multidisciplinary teams in tailoring design challenges, while virtual interactive gaze–based adaptive response technology [65] used real-time adaptive feedback to improve social communication. However, broader collaboration with policymakers and institutional stakeholders was rare, limiting scalability and integration into health care systems.

From a design perspective, these 2 forms of engagement can be understood as “design in the small” and “design in the large.” The former refers to participation in the artifact-level design (mechanics, interface, and content), whereas the latter concerns the contextual integration of the artifact within health care or educational systems. While most studies effectively address design in the small, few extend their focus to design in the large, leaving questions of implementation, sustainability, and policy alignment unresolved. Future research should therefore emphasize multilevel stakeholder engagement, involving decision-makers and regulatory partners to facilitate scaling from pilot projects to institutionalized practice [53].

### Summary

This review highlights distinct but interrelated design tendencies: (1) a preference for figural transfer and reflective boundary mechanisms, (2) a predominance of causal–procedural and relational–structural model clusters, and (3) a strong alignment between representational logic and learning mechanisms. These findings suggest that serious games for adolescent mental health are predominantly designed as reflective learning tools, fostering emotional and cognitive awareness rather than direct behavioral transformation. However, their impact depends less on which configuration is used and more on how deliberately these design choices are made and articulated. When the mechanisms of transfer, boundary crossing, and the model of reality are explicitly aligned within the design process, they can actively enable boundary crossing conditions.

From a design perspective, this alignment operates across 2 levels: design in the small, focusing on the internal coherence of the artifact (mechanics, narrative, fidelity, and user experience), and design in the large, emphasizing how the artifact interacts with its broader institutional, educational, or clinical context. By making these theoretical dimensions explicit during both levels of design, developers and researchers can create serious games that not only model health care processes but also facilitate real-world transfer and systemic learning.

## Discussion

### Principal Findings

This review highlights the transformative potential of serious games in health care while identifying key areas for improvement. A major challenge is the need for a coherent design rationale that explicitly integrates transfer, boundary crossing, and models of reality into the process of game development. Without such a foundation, serious games risk remaining isolated interventions rather than scalable tools for systemic change. This risk is reflected in the reviewed studies by the limited occurrence of transformation mechanisms (3/33 studies), the predominance of figural transfer (24/33 studies), and the concentration of stakeholder engagement at the artifact level rather than at the level of institutional implementation. Recent meta-analyses confirm that although serious games show positive effects on engagement and short-term outcomes, long-term validation and theoretical consistency remain limited [79].

### Learning Transfer

Among the reviewed studies, 24 employed figural transfer and 10 literal transfer, indicating that most serious games rely on symbolic or metaphorical learning rather than direct simulation. Figural transfer supports generalizable emotional and cognitive skills, whereas literal transfer offers task-specific realism. This emphasis on figural approaches suggests a preference for flexibility over precision but also highlights the need for scaffolding and debriefing mechanisms to reinforce learning. Drawing on educational and simulation-based research [80,81], future designs should integrate structured reflection and adaptive feedback to strengthen transfer beyond gameplay. This finding mirrors recent discussions in digital learning and simulation literature, emphasizing the importance of scaffolding mechanisms for durable learning outcomes [82,83].

Patterns in the data also reveal an alignment between transfer type and model typology: games employing causal or relational models tend to favor figural transfer. This finding suggests that representational logic and learning design interact closely, underscoring the importance of explicit design decisions to ensure coherence between game mechanics, learning goals, and therapeutic intent. This alignment is further illustrated by the absence of relational–literal pairings and by the concentration of figural transfer within causal and relational primary models (see Multimedia Appendix 2).

### Boundary Crossing

Boundary crossing was frequently observed but unevenly implemented. Reflection appeared in 22 studies, whereas transformation, representing deep cross-context learning, occurred in only 3 studies. Most games promoted perspective taking or self-awareness but stopped short of enabling collaborative learning or system-wide integration. This pattern corresponds with the low frequency of transformation as a boundary-crossing mechanism and suggests that learning is rarely designed to extend beyond the immediate intervention context. According to Akkerman and Bakker [29], transformation emerges when learning processes bridge distinct social or professional domains.

Explicitly defining intended boundary-crossing mechanisms within the design process could help serious games act as boundary objects, facilitating communication among clinicians, educators, and developers. This translational function positions games not only as therapeutic tools but also as mediators that enable shared understanding across sectors of health care.

### Models of Reality

Analysis of model types revealed a dominance of causal and procedural frameworks, with relational and structural models often appearing as secondary. These choices reflect a focus on mechanistic and process-oriented representations, supporting procedural learning that may limit the representation of social and ethical complexity when relational or structural models are not explicitly incorporated. This aligns with Bogost’s [38] notion of procedural rhetoric, where games express meaning through rules and system dynamics rather than narrative content.

While causal and procedural models effectively convey behavioral and process learning, they risk neglecting the interpersonal dimensions of health care. By contrast, relational and structural models, exemplified in iSocial [58] and CHARISMA-virtual social training [51], capture the social and contextual aspects of health practice. Integrating these model types can produce games that are both systematically rigorous and socially responsive, mirroring the real-world complexity of health care environments.

### Stakeholder Engagement and Design Scales

Stakeholder engagement was a consistent strength but remained concentrated at the artifact level. Across the reviewed studies, participatory involvement most frequently informed game mechanics, content, or usability, while engagement with organizational, policy, or stakeholders was rarely reported. Most studies prioritized design in the small through participatory methods involving therapists, educators, or caregivers, but few extended collaboration to policy, administration, or implementation levels. Expanding engagement to design in the large is essential for ensuring that serious games transition from prototypes to sustainable health care tools [84].

### Synthesis and Implications

Across studies, serious games for adolescent health care predominantly support reflective and representational learning, emphasizing awareness and regulation rather than long-term behavioral transformation. However, the findings indicate that effectiveness depends less on any single design dimension than on their explicit alignment.

When transfer mechanisms, boundary-crossing strategies, and model logics are purposefully coordinated, serious games can actively enable boundary-crossing conditions. This allows learning to flow between digital environments, clinical practice, and educational systems. Such alignment strengthens both the internal validity of the game as a learning artifact and its external applicability as a health care intervention.

Based on the observed design patterns across the reviewed studies, these implications can be understood as a conceptual synthesis rather than a prescriptive model. In practice, this means designing serious games as multilevel systems that bridge theory and implementation, ensuring fidelity, feedback, and learner engagement, embedding cross-professional collaboration and evaluation, and aligning design with institutional and policy frameworks. With this approach, future developers and researchers can create serious games that not only demonstrate short-term efficacy but also achieve sustained, scalable, and clinically relevant impact in health care delivery.

### Strengths and Limitations

This review advances serious game research by applying a structured multicriteria framework to examine design rationale, learning transfer, and real-world applicability. By analyzing 33 studies through the lenses of transfer, boundary crossing, and models of reality, this review provides one of the first quantified overviews of how theoretical constructs are applied in serious game design. The inclusion of typological and frequency analyses strengthens methodological transparency and highlights recurring design patterns.

However, several limitations should be acknowledged. The review is limited to published studies, introducing publication bias toward positive outcomes. While coding followed systematic criteria, some interpretive judgments were necessary to infer implicit design rationales. In addition, full PRISMA 2020 compliance and broader database coverage would enhance reproducibility. Finally, the scarcity of longitudinal studies constrains the understanding of sustained behavioral or cognitive impact. Despite these constraints, this review provides a solid foundation for future work. It shows that making design choices around transfer, boundary mechanisms, and models of reality explicit and aligned can help create more scalable, theoretically grounded health care games.

### Future Research

Future research should pursue longitudinal studies to evaluate how learning from serious games endures in real-world health care settings. Establishing standardized methods for assessing transfer, boundary crossing, and models of reality will improve comparability and design validation. Emphasis should be placed on explicit, theory-informed design alignment and interdisciplinary collaboration among developers, clinicians, and researchers. Broader stakeholder engagement, including policymakers, is vital for scaling interventions and integrating serious games into health care systems. These efforts can transform serious games from isolated innovations into sustainable, evidence-based tools that enhance learning, practice, and health care delivery.

### Conclusions

Building on these insights, this review demonstrates the transformative potential of serious games in health care, particularly for individuals with developmental and behavioral needs [1,43]. By fostering skill acquisition, collaboration, and meaningful transfer between digital and real-world contexts, serious games offer promising tools for advancing emotional regulation, cognitive development, and social interaction. However, their scalability and long-term impact remain limited by inconsistently articulated design rationales, which hinder replicability and adaptation across health care settings [29,35].

By systematically examining learning transfer, boundary crossing, and models of reality, this review provides an evidence-based framework for understanding how design decisions influence learning outcomes and real-world applicability. The findings highlight that explicit alignment among these constructs supported by participatory co-design and interdisciplinary collaboration is essential for achieving sustainable health care innovation. Broadening stakeholder engagement to include clinicians, caregivers, policymakers, and end users can further strengthen contextual integration and scalability [64].

Future research should focus on developing standardized, theory-informed frameworks [33], implementing longitudinal and mixed methods evaluations [44], and establishing implementation guidelines that connect design in the small (artifact-level) with design in the large (systemic integration). Through such structured, evidence-based approaches, serious games can evolve from isolated prototypes into validated, scalable instruments that enhance clinical decision-making, therapeutic engagement, and professional development in modern health care. Future work should also focus on validating these design principles across larger datasets and contexts. Comparative and meta-analytic approaches could test how alignment among transfer, boundary crossing, and model typology predicts real-world outcomes, advancing serious game design as an evidence-based methodology in health care.

### Key Takeaway

This review shows that serious games in adolescent health care predominantly support reflective and metaphorical learning, while rarely specifying how learning is expected to transfer beyond the game context. Making transfer mechanisms, boundary-crossing strategies, and underlying models of reality explicit during design could improve comparability, validation, and scalability of serious games in health care practice.

## Supplementary material

10.2196/77173 Multimedia Appendix 1 Search key appendix as referenced in the Methods.

10.2196/77173 Multimedia Appendix 2 Coding count and cross table.

10.2196/77173 Checklist 1 PRISMA 2020 guidelines.

## Abbreviations

PRISMA: Preferred Reporting Items for Systematic Reviews and Meta-Analyses
