# Supplementary material for: Research Design Processes in Serious Games for Adolescent Mental Health: Systematic Review
Source: JMIR Serious Games. 2026 Apr 10;14:e77173. doi: 10.2196/77173 (PMC13068190; doi:10.2196/77173)
Supplement: Multimedia Appendix 1 [file games-v14-e77173-s001.docx]

Appendix A, Search Keys

Initial key:

('health care'/exp OR healthcare:ab,ti,kw OR 'health care':ab,ti,kw OR (health NEAR/4 care)) AND ('adolescent'/exp OR (aged NEAR/3 (12 OR 13 OR 14 OR 15 OR 16 OR 17)) OR adolescent:ab,ti,kw OR teen*:ab,ti,kw OR youth:ab,ti,kw OR juvenile:ab,ti,kw) AND ('serious game'/exp OR 'serious gam*':ab,ti,kw OR 'game-based*':ab,ti,kw OR 'serious play':ab,ti,kw OR 'gamification':ab,ti,kw OR 'virtual realit*':ab,ti,kw OR 'vr':ab,ti,kw OR 'augmented realit*':ab,ti,kw OR 'ar':ab,ti,kw OR 'mixed realit*':ab,ti,kw OR 'mr':ab,ti,kw OR 'immersive technolog*':ab,ti,kw OR 'immersive experience*':ab,ti,kw OR 'simulated environment*':ab,ti,kw OR 'simulated experience*':ab,ti,kw) AND ('autism'/exp OR 'anxiety disorder'/exp OR 'anxiety'/exp OR 'attention deficit hyperactivity disorder'/exp OR autism:ab,ti,kw OR 'attention deficit disorder':ab,ti,kw OR add:ab,ti,kw OR adhd:ab,ti,kw OR anxiet*:ab,ti,kw OR 'attention deficit hyperactivity disorder':ab,ti,kw)

Embase search; recorded 13 april 2023

('adolescent'/exp OR (aged NEAR/3 (12 OR 13 OR 14 OR 15 OR 16 OR 17)) OR adolescent:ab,ti,kw OR teen*:ab,ti,kw OR youth:ab,ti,kw OR juvenile:ab,ti,kw) AND ('serious game'/exp OR 'virtual reality'/exp OR 'augmented reality'/exp OR 'serious gam*':ab,ti,kw OR 'game-based*':ab,ti,kw OR 'serious play':ab,ti,kw OR 'gamification':ab,ti,kw OR 'virtual realit*':ab,ti,kw OR vr:ab,ti,kw OR 'augmented realit*':ab,ti,kw OR ar:ab,ti,kw OR 'mixed realit*':ab,ti,kw OR mr:ab,ti,kw OR 'immersive technolog*':ab,ti,kw OR 'immersive experience*':ab,ti,kw OR 'simulated environment*':ab,ti,kw OR 'simulated experience*':ab,ti,kw) AND ('autism'/exp OR 'anxiety disorder'/exp OR 'anxiety'/exp OR 'attention deficit hyperactivity disorder'/exp OR autism:ab,ti,kw OR autistic:ab,ti,kw OR 'attention deficit disorder':ab,ti,kw OR add:ab,ti,kw OR adhd:ab,ti,kw OR anxiet*:ab,ti,kw OR 'attention deficit hyperactivity disorder':ab,ti,kw OR angst:ab,ti,kw OR anxiousness:ab,ti,kw)

Result: 1125 references

PubMed search; recorded 13 april 2023

((("Autism Spectrum Disorder"[Mesh] OR "Anxiety"[Mesh] OR "Anxiety Disorders"[Mesh] OR "Attention Deficit Disorder with Hyperactivity"[Mesh] OR autism[tiab] OR autistic[tiab] OR 'attention deficit disorder'[tiab] OR add[tiab] OR adhd[tiab] OR anxiet*[tiab] OR 'attention deficit hyperactivity disorder'[tiab] OR angst[tiab] OR anxiousness[tiab]) AND ("Video Games"[Mesh] OR "Augmented Reality"[Mesh] OR "Virtual Reality"[Mesh] OR "Gamification"[Mesh] OR 'serious gam*[tiab] OR 'game-based*'[tiab] OR 'serious play'[tiab] OR gamification[tiab] OR 'virtual realit*'[tiab] OR vr[tiab] OR 'augmented realit*'[tiab] OR ar[tiab] OR 'mixed realit*'[tiab] OR mr[tiab] OR 'immersive technolog*'[tiab] OR 'immersive experience*'[tiab] OR 'simulated environment*'[tiab] OR 'simulated experience*'[tiab])) AND ("Adolescent"[Mesh] OR adolescent*[tiab] OR teen*[tiab] OR youth[tiab] OR juvenile[tiab] OR "aged 12" [tiab:~3] OR "aged 13" [tiab:~3] OR "aged 14" [tiab:~3] OR "aged 15" [tiab:~3] OR "aged 16" [tiab:~3] OR "aged 17" [tiab:~3]))

Result: 984 references

PsychInfo; recorded 13 april 2023

( ( DE "Autism Spectrum Disorders" OR DE "Anxiety" OR DE "Anxiety Disorders" OR DE "Attention Deficit Disorder with Hyperactivity" OR DE "Attention Deficit Disorder" ) OR ( TI ( autism OR autistic OR 'attention deficit disorder' OR add OR adhd OR anxiet* OR 'attention deficit hyperactivity disorder' OR angst OR anxiousness ) OR AB ( autism OR autistic OR 'attention deficit disorder' OR add OR adhd OR anxiet* OR 'attention deficit hyperactivity disorder' OR angst OR anxiousness ) ) ) AND ( TI ( 'serious gam*' OR 'game-based*' OR 'serious play' OR 'gamification' OR 'virtual realit*' OR vr OR 'augmented realit*' OR ar OR 'mixed realit*' OR mr OR 'immersive technolog*' OR 'immersive experience*' OR 'simulated environment*' OR 'simulated experience*' ) OR AB ( 'serious gam*' OR 'game-based*' OR 'serious play' OR 'gamification' OR 'virtual realit*' OR vr OR 'augmented realit*' OR ar OR 'mixed realit*' OR mr OR 'immersive technolog*' OR 'immersive experience*' OR 'simulated environment*' OR 'simulated experience*' ) OR ( DE "Virtual Reality" OR DE "Augmented Reality" OR DE "Computer Games" ) ) AND ( TI ( adolescent* OR teen* OR youth OR juvenile ) OR AB ( adolescent* OR teen* OR youth OR juvenile ) OR ( aged NEAR/3 (12 OR 13 OR 14 OR 15 OR 16 OR 17) ) )

Result: 396 references

No DE (descriptors/keywords) for youth, adolescent or juvenile in psychinfo.

Eric search; recorded 19 april 2023

(DE "Autism" OR DE "Anxiety Disorders" OR DE "Anxiety" OR DE "Attention Deficit Disorders" OR DE "Attention Deficit Hyperactivity Disorder" OR TI ( autism OR autistic OR 'attention deficit disorder' OR add OR adhd OR anxiet* OR 'attention deficit hyperactivity disorder' OR angst OR anxiousness ) OR AB ( autism OR autistic OR 'attention deficit disorder' OR add OR adhd OR anxiet* OR 'attention deficit hyperactivity disorder' OR angst OR anxiousness ) ) AND ( ( DE "Computer Simulation" OR DE "Computer Games" OR DE "Game Based Learning" ) OR ( TI ( 'serious gam*' OR 'game-based*' OR 'serious play' OR 'gamification' OR 'virtual realit*' OR vr OR 'augmented realit*' OR ar OR 'mixed realit*' OR mr OR 'immersive technolog*' OR 'immersive experience*' OR 'simulated environment*' OR 'simulated experience*' ) OR AB ( 'serious gam*' OR 'game-based*' OR 'serious play' OR 'gamification' OR 'virtual realit*' OR vr OR 'augmented realit*' OR ar OR 'mixed realit*' OR mr OR 'immersive technolog*' OR 'immersive experience*' OR 'simulated environment*' OR 'simulated experience*' ) ) ) AND ( DE "Adolescents" OR DE "Youth" OR DE "Early Adolescents" OR TI ( adolescent* OR teen* OR youth OR juvenile ) OR AB ( adolescent* OR teen* OR youth OR juvenile ) OR ( aged NEAR/3 (12 OR 13 OR 14 OR 15 OR 16 OR 17) )

Result: 103 references

Scopus search; Recorded 19 april 2023

( TITLE-ABS-KEY ( "Autism Spectrum Disorders" OR anxiet* OR "Attention Deficit Disorder with Hyperactivity" OR "Attention Deficit Disorder" OR autism OR autistic OR add OR adhd OR "attention deficit hyperactivity disorder" OR angst OR anxiousness ) ) AND ( TITLE-ABS-KEY ( "serious gam*" OR "game-based*" OR "serious play" OR "gamification" OR "virtual realit*" OR vr OR "augmented realit*" OR ar OR "mixed realit*" OR mr OR "immersive technolog*" OR "immersive experience*" OR "simulated environment*" OR "simulated experience*" OR "Computer Game*" ) ) AND ( TITLE-ABS-KEY ( ( adolescent* OR teen* OR youth OR juvenile ) OR ( aged AND near/3 ( 12 OR 13 OR 14 OR 15 OR 16 OR 17 ) ) ) )

Result: 1389 references
