# Supplementary material for: Research Design Processes in Serious Games for Adolescent Mental Health: Systematic Review
Source: JMIR Serious Games. 2026 Apr 10;14:e77173. doi: 10.2196/77173 (PMC13068190; doi:10.2196/77173)
Supplement: Multimedia Appendix 2 [file games-v14-e77173-s002.docx]

## Appendix B, Cross-tab analysis tables.

Table 1. Frequency of learning mechanisms, transfer types, and model types (N = 33).

| Category | Count |
| --- | --- |
| Learning mechanism |  |
| Identification | 9 |
| Coordination | 13 |
| Reflection | 22 |
| Transformation | 3 |
| Transfer type |  |
| Figural | 24 |
| Literal | 10 |
| Model type (primary) |  |
| Structural | 5 |
| Causal | 12 |
| Relational | 9 |
| Procedural | 7 |
| Model type (secondary) |  |
| Structural | 8 |
| Causal | 7 |
| Relational | 6 |
| Procedural | 12 |

Table 2. Cross-tabulation of primary and secondary model of reality types (N = 33).

| Model type | Structural | Causal | Relational | Procedural |
| --- | --- | --- | --- | --- |
| Structural | x | 2 | 4 | 2 |
| Causal | 2 | x | 2 | 3 |
| Relational | 0 | 4 | x | 2 |
| Procedural | 3 | 6 | 3 | x |

Table 3. Cross-tabulation of primary model type and transfer type (N = 33).

| Transfer type | Literal | Figural |
| --- | --- | --- |
| Structural | 3 | 9 |
| Causal | 0 | 9 |
| Relational | 2 | 3 |
| Procedural | 4 | 3 |
